# The potential application of dual-energy subtraction radiography for COVID-19 pneumonia imaging

**DOI:** 10.1259/bjr.20201384

**Published:** 2021-03-18

**Authors:** Brent van der Heyden

**Affiliations:** 1 KULeuven, Department of Oncology, Laboratory of Experimental Radiotherapy, Leuven, Belgium

## Abstract

X-ray imaging plays a crucial role in the confirmation of COVID-19 pneumonia. Chest X-ray radiography and CT are two major imaging techniques that are currently adopted in the diagnosis of COVID-19 pneumonia. However, dual-energy subtraction radiography is hardly discussed as potential COVID-19 imaging application. More advanced X-ray radiography equipment often supports dual-energy subtraction X-ray radiography. Dual-energy subtraction radiography enables the calculation of pseudo-radiographs, in which bones are removed and only soft-tissues are highlighted. In this commentary, the author would like to draw the attention to the potential use of dual-energy subtraction X-ray radiography (*i.e.* soft-tissue pseudo-radiography) for the assessment and the longitudinal follow-up of COVID-19 pneumonia.

## Commentary

Chest X-ray radiography imaging plays a crucial role in the confirmation of pneumonia in patients with severe respiratory symptoms caused by COVID-19.^1^ X-ray radiography has already been suggested as first-line imaging modality of patients with suspected COVID-19 pneumonia in a pandemic scenario with a worldwide increasing number of inpatients.^2,3^ Alternatively, institutes adopted CT imaging as main or complimentary diagnostic tool for the assessment and the evaluation of COVID-19 pneumonia over time.^4–7^ Undoubtedly, CT imaging provides superior three-dimensional diagnostic information in contrast to planar X-ray radiography. Nevertheless, the CT imaging workflow requires more time, involves a much larger radiation dose,^8,9^ and cannot be followed locally at the bedside. These are important aspects that should normally be considered in a pandemic scenario where health-care workers and hospitals work under constantly increased pressure.

**Figure 1.**
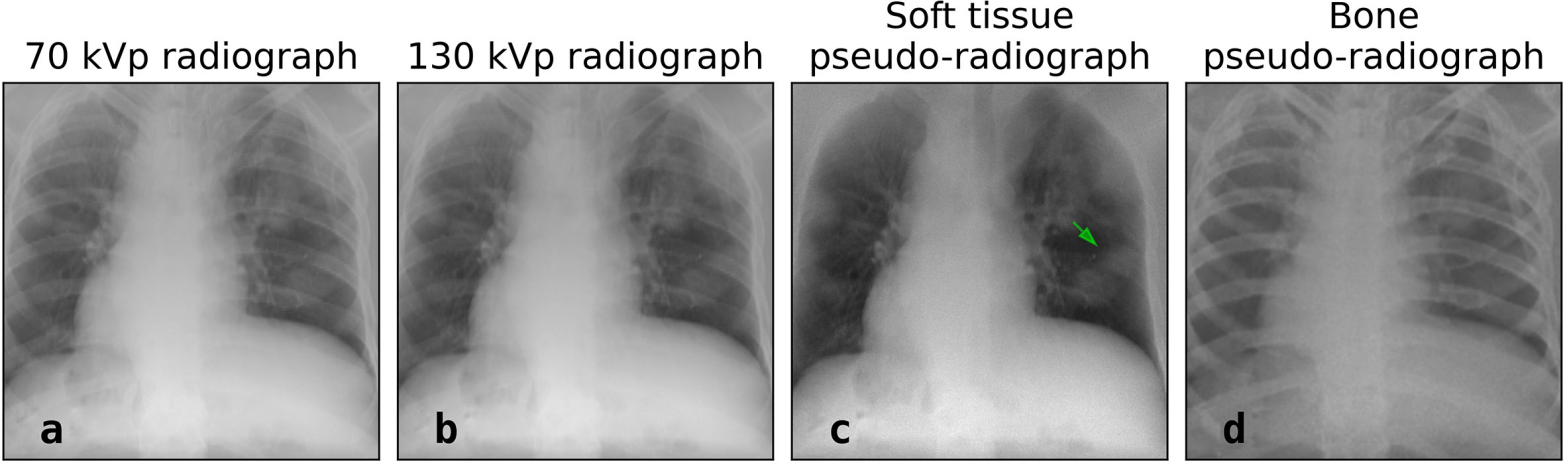
The low (a) and high (b) energy X-ray radiographs, and the post-processed pseudo-radiographs representing the soft-tissue (c) and the bone content (d) in the chest of a patient diagnosed with COVID-19 pneumonia. (Data set: coronacases_002.nii.gz from Jun et al^12^)

The author would like to draw attention to the potential use of dual-energy subtraction chest X-ray radiography in the assessment or evaluation of COVID-19 pneumonia. Dual-energy subtraction radiography is a well-established imaging technique available in several commercial devices that acquires two imaging exposures at different X-ray energies in a relatively short time-interval between the successive exposures (∼200 ms).^10,11^ Dual-energy radiography is performed at one low X-ray energy spectrum (*e.g.* 70 kVp) and at one high X-ray energy spectrum (*e.g.* 130 kVp). Therefore, dual-energy subtraction radiography takes advantages of the more pronounced X-ray attenuation changes at lower X-ray energies in calcium-containing human tissues such as cortical bone. Applying dedicated weighted post-processing techniques on two X-ray exposures facilitates the calculation of two separate pseudo-radiographs representing the (i) soft-tissue content, and the (ii) bone content (Figure 1c–d).

It should be noted that dual-energy subtraction radiography leads to an increased radiation dose compared to conventional radiography when the medical device is operating in dual-shot mode. However, the radiation dose of dual-energy subtraction X-ray radiography (∼0.2 mSv) is still substantially lower than the dose given by a chest CT scan (∼7.0 mSv).^9^ Additionally, image artifacts could be introduced near the diaphragm or cardiac wall due to respiratory or cardiac motion within the 200 ms exposure timeframe.^10^ Often, this effect is minimal and can be resolved by post-processing software.

Publications on bone suppression X-ray chest radiography (*i.e.* soft-tissue pseudo-radiographs) have shown increased sensibility in other subtle imaging applications, such as the recognition of pulmonary nodules and interstitial lung diseases.^13–15^ Therefore, it could also be relevant to validate the potential use of dual-energy subtraction X-ray imaging in the diagnosis of COVID-19 pneumonia.

To the author’s best knowledge, public dual-energy radiography data sets of COVID-19 pneumonia are currently lacking, and for that reason, realistically simulated radiographs are presented in this commentary as example. The dual-energy X-ray chest radiographs are simulated from an open-access high-resolution CT data set of a patient diagnosed with COVID-19 pneumonia.^12^ It is anticipated that the image quality of true pseudo-radiographs will outperform the quality of simulated radiographs due to computational restrictions. A detailed Monte Carlo simulation workflow is provided as Supplementary Material 1.

Supplementary Material 1.

Figure 1(a–b) presents the 70 kVp and 130 kVp chest X-ray radiographs, and Figure 1(c–d) shows the soft-tissue and bone pseudo-radiographs obtained after dedicated post-processing and dual-energy subtraction. The author foresees that the lung volume after bone suppression (Figure 1c), and thus COVID-19 pneumonia, could be better visible in soft-tissue pseudo-radiographs than in conventional X-rays chest radiographs. Especially in early stage COVID-19 pneumonia, the dense ribs could possibly mask essential anatomical information which would have stayed unnoticed in conventional X-ray radiography (*e.g.* Figure 1c, green arrow). The application of dual-energy subtraction radiograph has been confirmed in literature to provide superior soft-tissue pseudo radiographs in several radiological applications.^13,14,16^ Based on the available literature and the simulated imaging data presented in this commentary, it would be opportune to investigate the potential added value of soft-tissue pseudo-radiography in the diagnosis of COVID-19 pneumonia with prospective imaging trials.
