# Supplementary material for: The potential application of dual-energy subtraction radiography for COVID-19 pneumonia imaging
Source: Br J Radiol. 2021 Mar 18;94(1120):20201384. doi: 10.1259/bjr.20201384 (PMC8010552; doi:10.1259/bjr.20201384)
Supplement: Supplementary Material 1. [file bjr.20201384.suppl-01.pdf]

Supplementary Material: The Monte Carlo simulation of X-ray radiographs

The dual-energy X-ray chest radiographs were simulated with data from an open-access high-resolution CT dataset of patients diagnosed with COVID-19 pneumonia [1]. A Monte Carlo simulation platform was developed in TOPAS (V3.5, built on Geant4.10.06.p1) [2, 3]. The platform existed of three major components: the X-ray spectrum, the simulation geometry, and the CsI flat-panel imaging detector.

X-ray spectra

The 70 kVp and 130 kVp X-ray imaging spectra were calculated in SpekPy, an open-access software toolkit for the modelling of X-ray spectra [4]. In SpekPy, 0.15 mm Al and 0.20 mm Cu filtering were applied, and the X-ray tube anode angle was set to 10 degrees. The SpekPy X-ray spectra embedded in the Monte Carlo platform assumed a gaussian distributed focal spot with a size of 0.3 mm [5]. All simulations were performed with 80 billion histories.

Simulation geometry

The ‘coronacases_002.nii.gz’ dataset with 0.684 x 0.684 x 1.500 mm^3 voxel spacing was picked as digital anthropomorphic patient geometry [1]. The digital patient geometry was converted to a material map with unique tissue-density combinations. The Woodard and White tissues [6] were assigned by simple image thresholding in Hounsfield Units (HU), listed in Table 1.

Table 1. Material threshold values in Hounsfield Units (HU)

**Material**	**Minimum HU value**	**Maximum HU value**
Air	-1000	-900
Lung	-900	-200
Adipose	-200	0
Muscle	0	100
Cartilage Bone	100	400
Cortical Bone	400	1750

The CT scanner calibration curve from HU to mass density is unknown for this public dataset, and therefore, a simplified calibration curve with three datapoints was reproduced empirically. The radiodensity of air (0.0012 g/cm^3) and water (1 g/cm^3) are defined at -1000HU and 0 HU, the first two datapoints in the linear calibration curve. High-density bone (1.85 g/cm^3) was manually segmented in the CT dataset with ITK-SNAP [7] to obtain the third datapoint from the calibration curve. A mean HU value of 1750 HU was calculated in the segmented bone volume-of-interest. A total number of 2751 unique tissue-density combinations were used for simulation after applying the material thresholding and the CT number to mass density calibration curve.

Flat-panel imaging detector

A flat-panel imaging detector with an active area of 41 x 41 cm^2 was composed from mathematical volumes (TsBox) in the TOPAS platform. The detector model included a protective entrance carbon plate (1 mm), an air gap (~3 mm), a CsI scintillator (600 µm), a 1 mm glass substrate for the photodiodes, and an aluminum back plate (1.5 mm). Although submillimeter pixel sizes (< 0.2 mm) exist in modern flat-panel technology, 1 x 1 mm^2 pixel sizes are simulated to increase calculation efficacy of the Monte Carlo simulation model. A dedicated dose scorer was attached to the CsI scintillation layer, where the radiograph is formed.

References

1. Jun M, Cheng G, Yixin W, Xingle A, Jiantao G, Ziqi Y, et al. COVID-19 CT Lung and Infection Segmentation Dataset (Version 1.0). 2020.
2. Faddegon B, Ramos-Mendez J, Schuemann J, McNamara A, Shin J, Perl J, et al. The TOPAS tool for particle simulation, a Monte Carlo simulation tool for physics, biology and clinical research. Phys Med. 2020;72:114-21.
3. Perl J, Shin J, Schumann J, Faddegon B, Paganetti H. TOPAS: an innovative proton Monte Carlo platform for research and clinical applications. Med Phys. 2012;39(11):6818-37.
4. Bujila R, Omar A, Poludniowski G. A validation of SpekPy: A software toolkit for modelling X-ray tube spectra. Phys Med. 2020;75:44-54.
5. van der Heyden B, Fonseca GP, Podesta M, Messner I, Reisz N, Vaniqui A, et al. Modelling of the focal spot intensity distribution and the off-focal spot radiation in kilovoltage x-ray tubes for imaging. Phys Med Biol. 2020;65(2):025002.
6. Woodard HQ, White DR. The composition of body tissues. Br J Radiol. 1986;59(708):1209-18.
7. Yushkevich PA, Piven J, Hazlett HC, Smith RG, Ho S, Gee JC, et al. User-guided 3D active contour segmentation of anatomical structures: significantly improved efficiency and reliability. Neuroimage. 2006;31(3):1116-28.
